# Accuracy of insulin resistance indices for metabolic syndrome in a population with different degrees of glucose tolerance

**DOI:** 10.1186/1758-5996-7-S1-A115

**Published:** 2015-11-11

**Authors:** Luciana Pavan Antoniolli, Vanessa Piccoli, Mayara Abichequer Beer, Bárbara Limberger Nedel, Tássia Cividanes Pazinato, Lucas Eduardo Gatelli, Anize Delfino von Frankenberg, Monique de Moura Machado, Fernando Gerchman

**Affiliations:** 1 Universidade Federal do Rio Grande do Sul, Porto Alegre, Brazil

## Background

Insulin resistance has been associated with the development of metabolic syndrome (MS), which is an interrelated cluster of risk factors for cardiovascular disease and type 2 diabetes (T2D). Several equations derived from the oral glucose tolerance test (OGTT) have been developed as surrogates for the euglycemic hyperinsulinemic clamp technique to estimate insulin resistance and insulin sensitivity.

## Objectives

To determine the accuracy of insulin resistance (IRI) and the reciprocal of insulin sensitivity (ISI) indices to identify MS.

## Materials and methods

In a cross sectional study, subjects (n=183, females 73.2%; white color 82%; age 52.6±12.0; mean±SD) were submitted to a 2-h 75g OGTT (58 with normal glucose tolerance, 79 with prediabetes, 46 with T2D; ADA criteria). MS was classified according to IDF criteria (MS n=140, 76.5%). Glycosylated hemoglobin, adiponectin and lipid profile were tested. IRI was estimated by fasting insulin, fasting insulin/fasting glucose and 2h-insulin/2h-glucose ratios, FIRI, HOMA-AD, HOMA-IR, HOMA-2-IR and by the reciprocal of adiponectin, Avignon, Bennet, Gutt, HOMA-2-IS, ISi, ISi 2h, Matsuda, McAuley, QUICKI, Raynaud, Stumvoll and OGIS indices. The accuracy of IRI to identify MS was determined by ROC curve analysis and the identification of an optimal cut point was based on Youden index and distance to (0,1). It was considered p<0.001 for significant statistical differences in ROC curves comparison and p<0.05 in further analysis.

## Results

FIRI, HOMA-AD, HOMA-IR and the reciprocal of Avignon, Bennet, ISI, OGIS and QUICKI indices were directly related with fasting and 2h-plasma glucose, glycosylated hemoglobin, triglycerides levels, systolic and diastolic blood pressure (BP), waist circumference and body mass index, but they were inversely related with HDL-cholesterol. The reciprocals of Stumvoll and Gutt indices were also related with these variables, but not with diastolic BP. ROC analysis showed that the area under the curve was greater for 1/Gutt (0.864), 1/OGIS (0.828) and 1/Matsuda (0.790). By using an optimal cut point of 0.2680, 1/Gutt presented 86.4% sensitivity, 76.7% specificity, and a respective positive and negative likelihood ratio of 3.71 and 0.18 for MS.

## Conclusion

The reciprocal of the Gutt ISI was the most accurate method for assessing insulin resistance in a sample with a significant prevalence of MS and may be the preferred equation to estimate insulin sensitivity in subjects with MS.

